# Drug Development for Pediatric Populations: Regulatory Aspects

**DOI:** 10.3390/pharmaceutics2040364

**Published:** 2010-11-29

**Authors:** Jochen Zisowsky, Andreas Krause, Jasper Dingemanse

**Affiliations:** Actelion Pharmaceuticals Ltd, Clinical Pharmacology, Gewerbestrasse 16, CH-4123 Allschwil, Switzerland

**Keywords:** pediatrics, children, pediatric drug development, pediatric legislation, health authorities, regulatory guidelines, PIP, clinical studies, Modeling and Simulation, PK/PD modeling

## Abstract

Pediatric aspects are nowadays integrated early in the development process of a new drug. The stronger enforcement to obtain pediatric information by the regulatory agencies in recent years resulted in an increased number of trials in children. Specific guidelines and requirements from, in particular, the European Medicines Agency (EMA) and the Food and Drug Administration (FDA) form the regulatory framework. This review summarizes the regulatory requirements and strategies for pediatric drug development from an industry perspective. It covers pediatric study planning and conduct, considerations for first dose in children, appropriate sampling strategies, and different methods for data generation and analysis to generate knowledge about the pharmacokinetics (PK) and pharmacodynamics (PD) of a drug in children. The role of Modeling and Simulation (M&S) in pediatrics is highlighted—including the regulatory basis—and examples of the use of M&S are illustrated to support pediatric drug development.

## 1. Introduction

Over the past decade, regulatory legislations for drug development in pediatric patients were passed worldwide, dramatically increasing the number of drugs tested in and labeled for children. Both, the Food and Drug Administration (FDA) in the United States (U.S.), and the European Medicines Agency (EMA) in the European Union (E.U.), established approaches that have been successful in generating important new information about the safety and efficacy of drugs used by children [1,2]. Transparency and accountability of pediatric drug development has improved and the amount and quality of pediatric information was increased by an elevated number of clinical trials in children in recent years.

The progress was achieved by combining requirements for pediatric drug development with incentives for the pharmaceutical industry to (at least partly) cover the additional investment for testing drugs in children. There was and still is effort needed to harmonize the regulatory framework for pediatric drug development, but as of today pharmaceutical companies are still facing the problem that the regulatory requirements differ between FDA and EMA and that the development of a new drug in the pediatric population has to be in line with requirements from both authorities. Enforced by the authorities—in particular the EMA—pediatric aspects have to be integrated early in the development process of a new drug and the general strategy has to be part of the overall development program.

Drug development for pediatric patients is accompanied by various challenges for pharmaceutical companies. Clinical studies in children are different from studies in adults and the planning and conduct of a pediatric study needs special attention since the patient population is more vulnerable. Practical and ethical considerations are prominent and the latter receives particular attention in the regulatory guidance documents. As a consequence, the main challenges are to define the first dose in children, to find appropriate sampling strategies, to choose the right methods for data collection and analysis, to generate knowledge about safety, efficacy, pharmacokinetics, and pharmacodynamics of a drug in children, and ultimately to determine the right dose and dosing regimen.

To comply with regulatory requirements and to optimize pediatric drug development—in terms of ethical, operational, and scientific aspects—pharmaceutical companies have to develop new and appropriate strategies for clinical studies in children. Modeling and Simulation (M&S) techniques are beneficial tools for optimization of the design, increasing the knowledge gained from pediatric studies. Since several regulatory guidance documents refer explicitly to M&S methodology, there is a clear regulatory basis and a need for using M&S in pediatric drug development.

This review gives an overview of the regulatory aspects for pediatric drug development and their influence on practical and scientific considerations when conducting clinical studies in children. M&S as an efficient means to extract knowledge from the data will be discussed subsequently, rounding off the process of pediatric study planning, conduct, and evaluation.

## 2. Regulatory Aspects of Pediatric Drug Development

### 2.1. U.S. perspective

Historically, only a small fraction of all marketed drugs have had clinical trials performed in pediatric patients and a majority of marketed drugs were not labeled for use in pediatric patients. Accordingly, many drugs were administered to children in an off-label fashion without adequate understanding of appropriate dose, safety, or efficacy [3].

The first initiative took place in 1994 when the Pediatric Labeling Rule was issued [4], requiring drug manufacturers to survey existing data and to determine whether those data are sufficient to support additional pediatric use information in the drug’s labeling. Under the Pediatric Labeling Rule, if a manufacturer determines that existing data permit modification of the label’s pediatric use information, the manufacturer must submit a supplemental new drug application (NDA) to FDA seeking approval of the label change [5]. The Pediatric Labeling Rule allowed the labeling of drugs for pediatric use based on extrapolation of efficacy in the adult population and additional pharmacokinetics, pharmacodynamics, and safety studies in pediatric patients, but only if the course of the disease and the response to the drug were known to be similar in children compared to adults. Although this rule was designed to improve pediatric labeling, only a small number of well-designed and well-conducted studies subsequently resulted [6].

Since the approach under the Pediatric Labeling Rule was entirely voluntary, and did not substantially increase the number of products with adequate pediatric labeling, the FDA proposed (1997) and finalized (1998) the *Pediatric Rule* [5,7]. The rule was designed to ensure that new drugs and biological products that are likely to be commonly used in children, or that represent a meaningful therapeutic benefit over existing treatments for children, contain adequate pediatric labeling for the approved indication at the time of, or soon after, approval. The rule would require a manufacturer of a new drug to submit, before approval, safety and effectiveness information in relevant pediatric age groups for the claimed indications. The submission of information could be deferred, e.g., if pediatric studies should not begin until information on adults had been collected, or in case the collection and filing of pediatric data would delay the availability of a product that provides a significant therapeutic advantage in adults. The requirement would be waived for some or all pediatric age groups if, e.g., the product did not represent a meaningful therapeutic benefit over existing treatment or the product would likely be unsafe or ineffective in pediatric patients [8].

Also in 1997, the Food and Drug Administration Modernization Act (FDAMA) introduced a process in which the FDA would develop a list of drugs for which additional pediatric information might be beneficial, agree on necessary studies, and issue to sponsors a Written Request (WR) for pediatric studies [9]. The WR includes a timeframe for completing such studies. In addition, the FDAMA provided an incentive for pharmaceutical companies to study products which would yield a health benefit in the pediatric population. If companies submitted studies responding to a WR, six additional months of marketing exclusivity were granted [10]. Many drugs have received pediatric labeling under this provision, such that the FDAMA could be considered as the major legislative initiative that progressed pediatric drug development in the U.S.

In 2001, the FDA's Report to Congress identified some drawbacks, for example, that the incentive legislation was only applicable to some drugs [11]. These drawbacks were partially addressed by the Best Pharmaceuticals for Children Act (BPCA) in 2002 [12]. The BPCA renewed the exclusivity incentives, created a process for on- and off-patent drugs involving government contracts for pediatric studies, and mandated public disclosure of study results [6].

In 2003, the Pediatric Research Equity Act (PREA) was enacted, putting into legislation most components of the Pediatric Rule. It required pediatric assessment for certain applications unless waived or deferred and a pediatric plan that outlines the pediatric assessment (including timelines) and addressed development of an age-appropriate formulation [13,14].

In summary, there are two separate legislations for pediatric drug development in the U.S.: the PREA defining the requirements and the BPCA defining the incentives. The PREA covers drugs and biologics and the studies are mandatory (only for indications under review, exempting orphan indications), whereas the BPCA covers only drugs and the studies are voluntary, relate to entire moiety, and might expand indications (including orphan indications). PREA and BPCA request pediatric studies to be labeled and pediatric safety data to be presented publicly to an advisory committee one year after study conduct. Both acts are clearly designed to encourage more pediatric research and more development of pediatric medicines.

In 2007, within the scope of the Food and Drug Administration Amendments Act (FDAAA), the PREA and the BPCA were amended and reauthorized [15]. The reauthorization extended the BPCA incentive and the PREA authority until October 2012 (Table 1). In addition, the FDAAA introduced the Pediatric Review Committee (PeRC). The PeRC includes employees of the FDA with expertise in pediatrics, clinical pharmacology, statistics, chemistry, legal issues, pediatric ethics, and appropriate expertise pertaining to the product under review as well as other designated individuals. The PeRC provides the framework for the preparation of consultation on and general review of pediatric information in pediatric plans, assessments, and pediatric studies to help ensure quality and consistency. The PeRC reviews all WRs, all deferrals and waivers, and submitted studies in response to a WR [16].

**Table 1:** Major milestones of pediatric legislation in the U.S.

1994	1997	2002	2003	2007
Pediatric Labeling Rule	Pediatric Rule FDAMA: Food and Drug Administration Modernization Act	BPCA: Best Pharma-ceutical For Children Act	PREA: Pediatric Research Equity Act	FDAAA: Food and Drug Administration Amendments Act

### 2.2. E.U. perspective

Similar to the U.S., the European Medicines Agency (EMA) perceived the need for legal obligations for pharmaceutical companies to perform pediatric studies to obtain pediatric information for medicines used in children. 

In 1997, the European Commission organized a round table of experts to discuss pediatric medicines at the EMA. The experts identified the need to strengthen the legislation, in particular by introducing a system of incentives [1].

In 1998, the Commission supported the need for international discussion on the conduct of clinical trials in children in the context of the International Conference of Harmonisation (ICH) [1]. In 2000, the harmonized tripartite E11 ICH guideline "Clinical investigation of medicinal products in the paediatric population" was finalized and subsequently became a European guideline in 2001 [1,17].

In December 2000, the European Health Council requested the Commission to take specific action to remedy the problem of usage of unauthorized medicinal products in the pediatric population [18]. One of the first steps of the Commission to address the problem was a consultation paper "Better medicines for children—proposed regulatory actions on paediatric medicinal products" (2002) [19]. 

In subsequent years, these proposals were assessed and resulted in a new legislation governing development and authorization of medicines for pediatric use. It was introduced in the European Union (E.U.) in December 2006 and entered into force January 2007 [20] (Table 2).

**Table 2:** Major milestones of pediatric legislation in the E.U.

1997	1998	2000	2002	2006	2007
EMEA Round Table	ICH Discussion	Guideline ICH E11	Consultation Paper	Pediatric Regulation Agreed	Pediatric Regulation Into Force

#### Main Pillars of E.U. regulation

In general, the objective of the E.U. regulation is to improve quality and ethical research into medicines for children, increase the availability of authorized medicines for children, and to increase available information on medicines for children without unnecessary studies in children and without delaying authorization for adults [21,22]. To achieve these objectives, the E.U. pediatric regulation is based on the following pillars:

*Pediatric Committee (PDCO) [23,24]:*The PDCO (speak: "pedco") is the counterpart to the PeRC in the U.S. It is a committee of experts with competence in development and assessment of all aspects of pediatric medicinal products: five members (and alternates) of the Committee for Medicinal Products for Human Use (CHMP), one member (and alternate) from each Member State not represented via CHMP membership, and six members (and alternates) appointed by the European Commission representing healthcare professionals and patients’ organizations. The main responsibility of the PDCO is to assess the content of submitted Pediatric Investigation Plans (PIP) and adopt opinions on them in accordance with the E.U. pediatric regulation. This includes the assessment of applications for a full or partial waiver and for deferrals. Other tasks of the PDCO include assessing data generated in accordance with the PIP, advising and supporting the EMA on creation of a European pediatric network, and establishing and regularly updating an inventory of pediatric medicinal needs.

*Pediatric Investigation Plan (PIP) [25]:* A PIP is the basis for development and authorization of a medicinal product for the pediatric population subsets (*i.e.*, the different age groups, see Table 3). It has to be submitted upon availability of adult PK studies, *i.e.*, at an early phase of development of a new compound (after Phase 1). The PIP has to be agreed upon and/or amended by the PDCO and is binding for the company. If new information becomes available during the development, it is possible to apply to the PDCO for a modification of the agreed-upon PIP.

**Table 3:** Classification of pediatric age categories [17,26].

ICH Guideline E11		FDA	
**Preterm newborn infants**		-	
**Term newborn infants:**	0 to 27 days	**Neonate:**	Birth to 1 month
**Infants and toddlers:**	28 days to 23 months	**Infant:**	1 month to 2 years
**Children:**	2 to 11 years	**Children:**	2 to 12 years
**Adolescents:**	12 to 16-18 years*	**Adolescent:**	12 to <16 years
* dependent on region			

The PIP includes details of the timing and the measures proposed to demonstrate quality, safety, and efficacy in the pediatric population and should cover all ages from birth to adolescence. It reflects the development plan in clinical, non-clinical, and technical aspects including timelines and covers all existing or planned (adult) indications and dosage forms (including specific age-appropriate pediatric formulations or routes of administration if necessary). The PIP clearly defines timing of studies in children relative to adults, including deferrals until completion of studies in adults to ensure that studies in children are conducted only when it is safe and ethical to do so. 

A waiver can be granted for some or all age groups if the drug is likely to be ineffective or unsafe in part or all of the pediatric population, if it is intended for conditions that occur only in adult populations (e.g., Alzheimer's disease), or if it does not represent a significant therapeutic benefit over existing treatments for pediatric patients.

The EMA provides on its homepage detailed information about the PIP procedure including a guideline on format and content of PIPs [25]_._ Information about EMA decisions on PIPs (and waivers) is made public after deletion of commercially confidential data.

*Rewards and incentives [20,21]:* It is mandatory to submit pediatric data in accordance to an agreed PIP for all regulatory submissions for new products and for products still on patent in case of line extension requests (unless a waiver or deferral was granted). Once authorization is obtained in all E.U. Member States and study results are included in the product information, also if results are negative, the medicine is eligible for six months of patent extension. Orphan-designated medicinal products are subject to the same requirements and benefit from two years of market exclusivity in addition to the 10-year exclusivity rewarded under the E.U. Orphan Regulation. A prerequisite for getting any incentive (and to receive marketing authorization) is a compliance check with the agreed PIP.

*Other tools for information, transparency, and stimulation of research [20,21]:* To improve the information available for medicines used in children, the EMA established an inventory of pediatric needs in the different therapeutic areas where research and development of medicinal products for children is needed [27]. As a transparency measure, all pediatric clinical studies performed in the E.U. are registered with the E.U. database on clinical trials (EudraCT), including all worldwide studies in children if the study is part of a PIP. Details of the results of pediatric clinical trials, including those terminated prematurely, will be publicized by the EMA [28].

For any pediatric development question, the EMA offers free scientific advice or protocol assistance (for orphan or rare diseases). The advice can be requested before submitting a PIP or after PDCO decision. The scientific advice is not binding for any PDCO decision [29].

Another objective of the E.U. regulation is to foster high quality ethical research on medicinal products to be used in children. To meet this objective, the establishment of a European Pediatric Research Network (EnprEMA) of existing national and European networks is required. As an outcome of the second workshop of the EnprEMA in March 2010, the structure of operation was defined by establishing a coordination group and recognition criteria for existing networks to become a member of the EnprEMA [30].

### 2.3. Comparison of U.S. (FDA) and E.U. (EMA) regulations

The primary goal of the E.U. and U.S. legislations is identical: to improve children's health through advancements in research and to provide a framework for evaluation of efficacy and safety in the pediatric population. The U.S. and the E.U. legislation show substantial differences though.

The E.U. legislation unifies the incentives and requirements under one legislation and the changes occurred in a shorter time frame: since July 2008 (18 months after entry into force), all applications for new marketing authorization must contain results of studies conducted in compliance with an agreed PIP unless a waiver or deferral was granted; since January 2009 (24 months after entry into force) all applications for new indications, new routes of administration, or new pharmaceutical forms must contain results of studies in compliance with the PIP unless a waiver or deferral was granted.

Moreover, the E.U. legislation is leading to more profound changes. In the E.U., pediatric development is mandatory for all new medicinal products under development unless a waiver is granted, and the pediatric product development is discussed earlier in the regulatory process compared to the U.S., *i.e.*, companies have to submit a PIP upon availability of adult PK studies. The PeRC and the PDCO have similar responsibilities, mainly to review the WR and the PIP, respectively. The PDCO has more authority though since its decisions are binding. 

The WR and the PIP differ as well. The WR is voluntary and issued by the FDA, usually following a proposed pediatric study request (PPSR) from the sponsor. The PIP is mandatory and proposed by the sponsor. The PIP addresses non-clinical requirements, complete product quality including age‑appropriate formulation, and includes waiver and deferral requests, whereas the WR includes age‑appropriate formulations statements, might include non-clinical studies, and does not include a waiver or deferral [2,22].

These differences are largely due to the two legislations in the U.S.: the PIP in the E.U. covers both the requirements and the incentives, whereas in the U.S., the incentives are covered by the WR (under the BPCA) and the requirements by the pediatric plan (under PREA).

Because of these differences, the FDA and the EMA developed a pediatric collaboration with exchange of information to avoid exposing children to unnecessary trials, to enhance the science and decrease the risk to children during pediatric drug development [31]. This communication does not imply that pediatric development programs will have exactly the same protocols or objectives or arrive at the same regulatory decisions, but it is a welcomed and important step towards a harmonized regulatory framework for pediatric drug development.

### 2.4. Japanese perspective

With Japan as part of the ICH, the tripartite harmonized ICH E11 guideline ''Clinical investigation of medicinal products in the pediatric population" [17] is the primary guideline for pediatric drug development in Japan. In addition, Japan joined the FDA and EMA collaboration and exchange as an observer [22].

### 2.5. Regulatory evaluation of pediatric legislation

It is of public interest to trace the pediatric legislation and its impact for the pharmaceutical industry. Therefore, the FDA and the EMA regularly evaluate their pediatric legislations and make the results publicly available. 

#### 2.5.1. EMA

The most recent EMA results are published in the "Report to the European Commission: Companies and products that have benefited from any of the rewards and incentives in the paediatric regulation and the companies that have failed to comply with any of the obligations in this regulation covering the years 2007 to 2009." (April 27, 2010) [32].

Between August 2007 and December 2009, the PDCO received 629 validated PIP applications (for 961 indications, on average 1.5 indications per product) of which 156 (25%) included requests for a full waiver for all conditions and all subsets of the pediatric population.

Since its first meeting in July 2007, the PDCO adopted 125 positive opinions on product-specific waivers (36%), 205 positive opinions on a PIP (59%), including deferrals and/or partial waivers, and 17 negative opinions (5%). Details of PDCO decisions are published regularly on the EMA webpage [33].

For the EMA, it is anticipated that the number of requests for modifications of an agreed PIP will increase exponentially. As (pediatric) drug development is a dynamic process depending on results of ongoing studies, it is anticipated that three to five modifications will be submitted per agreed-upon PIP. As of December 2009, the PDCO adopted 59 positive opinions on modifications of an agreed‑upon PIP [31].

#### 2.5.2. FDA

The FDA publishes statistics about pediatric exclusivity and pediatric studies on the FDA webpage [34]. The “Pediatric Exclusivity Statistics” show that as of the end of August 2010, the FDA received 610 Proposed Pediatric Study Requests (PPSRs) and issued 394 Written Requests (320 with PPSR and 74 without PPSR). FDA granted pediatric exclusivity for 173 approved drugs so far (end of August 2010) [35].

Between September 2007 and June 2010, 273 studies were completed under the BPCA (51), the PREA (151), or both (71) pursuant to the FDAAA. Efficacy and safety studies (178) and PK and safety studies (49) were most frequent [36]. The total number of products under BPCA and PREA (amended by FDAAA) is 38 and 65, respectively (end of August 2010) [37].

Although these results are based on different legislations and thus difficult to compare, both evaluations show clearly that the pediatric legislations overall have been very successful and will further increase the number of drugs tested and labeled for children.

### 2.6. Specific regulatory guidelines and guidance documents

Several specific regulatory guidelines for pediatric drug development have been released by the FDA and the EMA in recent years. These guidelines form the regulatory framework for the pharmaceutical industry when studying drugs in children. Table 4, Table 5 and Table 6 present an overview of the most important FDA and EMA regulatory pediatric guidelines and guidance documents. They can be categorized into procedural guidelines (Table 4), scientific guidelines (Table 5), and other guidelines with an impact on pediatric drug development (Table 6). The numerous documents reflect the need of the pharmaceutical industry to obtain scientific and procedural guidance to fulfill the quite complex and diverse regulatory requirements. It can be assumed that the number will increase further with increasing experience in pediatrics on both sides, the regulatory agencies and the pharmaceutical industry.

**Table 4:** Procedural guidelines for pediatric drug development.

**FDA Pediatric Guidelines**
• Recommendations for Complying with the Pediatric Rule (Draft Guidance) [8]
• How to Comply with the Pediatric Research Equity Act (Draft Guidance) [14]
• Qualifying for Pediatric Exclusivity Under Section 505A of the Federal Food, Drug, and Cosmetic Act [10]
• The Content and Format for Pediatric Use Supplements [4]
**EMA Pediatric Guidelines**
• Guideline on the format and content of applications for agreement or modification of a pediatric investigation plan and requests for waivers or deferrals and concerning the operation of the compliance check and on criteria for assessing significant studies [25]
• Guideline on the information concerning pediatric clinical trials to be entered into the EU Database on Clinical Trials (EudraCT) and on the information to be made public by the European Medicines Agency (EMEA) [27]

**Table 5:** Scientific guidelines for pediatric drug development.

**International Guidelines**
• Note for Guidance on Clinical Investigation of Medicinal Products in the Pediatric Population (ICH, E11) [17]
**FDA Pediatric Guidelines**
• General Considerations for the Clinical Evaluation of Drugs in Infants and Children [38]
• General Considerations for Pediatric Pharmacokinetic Studies for Drugs and Biological Products [26]
• Nonclinical Safety Evaluation of Pediatric Drug Products [39]
• Guidelines for the Clinical Evaluation of Antiepileptic Drugs (Adults and Children) [40]
• Orally Inhaled and Intranasal Corticosteroids: Evaluation of the Effects on Growth in Children [41]
• Guidelines for the Clinical Evaluation of Psychoactive Drugs in Infants and Children [42]
• Pediatric Oncology Studies in Response to a Written Request (Draft Guidance) [43]
**EMA Pediatric Guidelines**
• Guideline on the role of pharmacokinetics in the development of medicinal products in the pediatric population [44]
• Guideline on the investigation of medicinal products in the term and preterm neonate [45]
• Guideline on conduct of pharmacovigilance for medicines used by the pediatric population [46]
• Note for guidance on evaluation of anticancer medicinal products in man - Addendum on pediatric oncology [47]
• Clinical evaluation of medicinal products in weight control - Addendum on weight control in children [48]
• Guideline on the need for non-clinical testing in juvenile animals of pharmaceuticals for pediatric indications [49]
• Concept paper on the impact of brain immaturity when investigating medicinal products intended for neonatal use [50]
• Concept paper on the impact of liver immaturity when investigating medicinal products intended for neonatal use (draft) [51]
• Concept paper on the impact of lung and heart immaturity when investigating medicinal products intended for neonatal use (draft) [52]
• Pediatric addendum to the CHMP guideline on the clinical investigations of medicinal products for the treatment of pulmonary arterial hypertension (draft) [53]
• Pediatric addendum to the CHMP note for guidance on clinical investigation of medicinal products in the treatment of lipid disorders (draft) [54]
• Concept paper on the need for the development of a pediatric addendum to the note for guidance on the clinical investigation on medicinal products in the treatment of hypertension [55]
• Ethical considerations for clinical trials on medicinal products with the pediatric population [56]
• Discussion paper on the impact of renal immaturity when investigating medicinal products intended for pediatric use [57]
• Reflection paper: formulations of choice for the pediatric population [58]

**Table 6:** Other guidelines with impact on pediatric drug development.

**FDA Guidelines**
• Population Pharmacokinetics [59]
• Exposure-Response Relationships - Study Design, Data Analysis, and Regulatory Applications [60]
**EMA Guidelines**
• Guideline on clinical trials in small populations [61]
• Guideline on data monitoring committees [62]

## 3. Pediatric Study Planning and Conduct

Clinical studies in children differ from studies in adults in many respects. The diversity of children in different age groups, the consent and recruitment process or the ethical implications are only some examples to explain why the planning and conduct of a pediatric study needs particular attention.

### 3.1. General considerations

As soon as the decision is taken to develop a drug for a disease in adults that may have applicability in pediatrics, the drug development team should outline its strategy for pediatrics, answering the following three key questions [63]:

- Does the disease affect children?
- Is the disease / disease progression in children similar to that in adults?
- Is the outcome of therapy likely to be similar to that in the adult form of the disease?

If all of these three questions are answered by yes, pharmacokinetic data in children (together with appropriate safety data) may be sufficient to support an application for pediatric labeling without the need for extensive efficacy trials. In many cases though, these conditions are not met or sufficient information about a new drug is not available to answer these questions and clinical efficacy trials may be required. The same applies if the relationship between concentration and response is anticipated to be different in children compared to adults.

It is desirable to make maximum use of extrapolation to children of available adult efficacy data. The approach is acceptable if similarity in indication, mechanism or course of disease and outcome of therapy (both beneficial and adverse) is sufficient. The FDA describes this approach in the pediatric study decision tree: at minimum a PK / safety study would be sufficient to bridge between children and adults [60].

For most new drugs, however, it is challenging to predict if the concentration-response relationship in pediatric patients is similar to adults and to have a validated and accepted PD variable to predict efficacy. In practice, a PK bridging study is possible, but for a new compound it is often not realistic and PK, PD, and safety / efficacy information should be collected.

On outlining the general pediatric strategy, there are various other aspects a company has to consider before planning pediatric studies [17].

First of all, the type of disease being treated has to be a relevant pediatric disease and the appropriate risk/benefit assessment of engaging in pediatric trials has to take place, including considerations of efficacy and safety of alternative treatments. After defining the type and timing of pediatric studies, the assessment of the need for pediatric formulations for all appropriate age ranges will be an important step [58]. If no biomarkers or surrogate endpoints are available, the company may consider investigating or developing them. 

In order to design a clinical study in the pediatric population, preclinical aspects such as preclinical safety assessment and preclinical drug metabolism and pharmacokinetics are important. Data from rodent and non-rodent species can identify target organ toxicity and provide appropriate margins of safety between therapeutic exposure and those that produce adversity in non-clinical studies. Juvenile animal studies are an extension to this paradigm in providing a comparison between adults and immature forms of the animal species [39,49].

For planning and performing pediatric clinical trials, it is important to realize that such trials might take longer since recruitment may be much more difficult. In addition, clinical centers with pediatric expertise are needed for study conduct. Advice on design and conduct can be given by the EMA (free of charge) upon request prior to submission of a PIP or at a later stage [28].

### 3.2. Age categories

The proposed age categories (by ICH and FDA, see Table 3) are to some extent arbitrary, wide in terms of body weight, and may include different maturation levels or considerable developmental overlap. Accordingly, the adoption of flexible approaches is required to ensure that study design reflects current knowledge of pediatric pharmacology and developmental biology.

### 3.3. Determination of pediatric dose

In the past, the pediatric dose calculation was often based on a fraction of the adult dose. Scaling parameters such as mg/kg of body weight or mg/m^2^ of body surface area were commonly used [64]. Allometric scaling (for example the "3/4 Rule") was used frequently to extrapolate the adult clearance (or dose) to children [65]. All scaling approaches are simple, easy, and quick and—dependent on the drug—might give a good estimate of the pediatric dose. In particular for adolescents, dose scaling, e.g., based on body weight, frequently produces reasonable results as the developmental differences to adults are generally not substantial. However, simple scaling does not account for ontogeny of drug metabolizing enzymes in neonates and infants and, therefore, should only be used to normalize clearance / dose for children older than two years [66,67]. Even for children above two years of age, modifications of these initial estimates might be needed based on known adult ADME characteristics, any prior pediatric experience, and the physiological development of the intended pediatric study population.

M&S techniques such as population PK/PD modeling and physiologically based pharmacokinetic (PBPK) modeling can provide substantial support to determine the pediatric dose. 

When defining a dose for the pediatric population, in particular the dose to be administered initially, one should always recall that children are not small adults. The use of pharmacology/physiology knowledge together with the PK/PD relationship in adults and knowledge from drugs of the same pharmacological class is essential for selecting the first dose in pediatrics. The aim is to calculate the dose that provides the same systemic exposure as in adults taking into consideration the higher inter‑patient pharmacokinetic variability observed in children compared to adults [68], assuming the PK/PD relationship holds (similar exposure to drug yields similar pharmacodynamic response).

Furthermore, to retrieve the starting dose, the predicted therapeutic dose should be scaled by an appropriate safety factor. An approach could be to start dosing a small cohort (e.g., n = 4 to 6) and analyze safety, PK, and/or PD results. If this first dose is safe, a PK- and/or PD-guided dose escalation could be performed to reach target exposure and response. Once the target is reached, the cohort sample size can be increased to collect more data on safety, PK, and PD and/or efficacy.

With increased age, children become more similar to adults. Thus, if several studies with different age ranges are planned in the pediatric population, it is good advice to start with the older age groups and use the collected data adaptively to possibly modify the doses for the subsequent studies in the younger age groups.

Dependent on the drug, an individualization of therapy following initial dosing and careful observation of response or safety may be useful. A therapeutic drug monitoring of plasma concentrations should be considered if an exposure-response relationship is to be characterized.

### 3.4. PK sampling and PK evaluation in pediatrics

The EMA guideline "Ethical considerations for clinical trials on medicinal products with the paediatric population" [56] clearly states that the blood sampling volume related to the trial has to be minimized and justified in the protocol. In general, blood loss should not exceed 3% of total blood volume over four weeks, and it should not exceed 1% of total blood volume at any single time. Table 7 shows the acceptable blood volumes for different age ranges.

**Table 7:** Age ranges and corresponding volume limits for blood sampling [69,70].

	Whole blood volume (mL/kg)	Mean body weight (kg)	Whole blood volume (mL)	3% (mL)	1% (mL)
**Newborn, 1 day**	83	3.45	287	8.6	2.9
**Infant, 3 months**	87	6.15	535	16.1	5.4
**Infant, 6 months**	86	7.85	675	20.3	6.7
**Infant, 12 months**	80	10.1	808	24.2	8.1
**Children, 6 years**	80	20.6	1648	49.4	16.5
**Children, 10 years**	75	32.6	2445	73.4	24.5
**Adolescent, 15 years**	71	54.3	3855	115.7	38.6

The FDA provides no guidance on blood volume but describes that volume and frequency of blood sampling can be minimized by using micro-volume drug assays and sparse sampling techniques, respectively. In addition, non-invasive sampling procedures such as urine and saliva collection may suffice if the correlation with blood and/or plasma levels has been documented [38].

The ICH guideline E11 gives additional practical considerations with respect to PK blood sampling. Besides using a sensitive assay to decrease blood volume per sample (e.g., dried blood spot analysis), the use of experienced laboratories that can handle small blood volumes for PK and laboratory safety samples is recommended. Whenever possible, PK and laboratory safety samples should be collected at the same time to avoid repeat procedures. To minimize distress, the use of indwelling catheters rather than repeated venipunctures for blood sampling is recommended [17].

For optimized evaluation, several guidelines recommend the use of population PK modeling and sparse sampling to minimize the number of samples obtained from each patient (see Table 8). This includes sparse sampling approaches in which each patient contributes as few as two to four samples at predetermined times and population PK analysis using the most useful sampling time points (from a statistical, information-theoretic perspective) derived from modeling of adult data.

### 3.5. Pediatric formulations

The oral route of administration is commonly used for dosing to children and, therefore, many medicines should be available in both, liquid and solid oral dosage forms, in order to target a wide age range. Liquid formulations, for instance, are most appropriate for younger children who are unable to swallow capsules or tablets. Parenteral formulations are commonly used in neonates and extra care should be taken with respect to drug concentrations and choice of excipients.

The EMA requires that the PIP describes any measures to adapt the formulation of the medicinal product to make its use more acceptable, easier, safer, or more effective for different subsets of the pediatric population. The EMA reflection paper "Formulations of choice for the paediatric population" [58] is not intended as a regulatory guidance document that defines requirements to be fulfilled. It provides helpful suggestions and has been written to summarize available information on pediatric formulations and to use examples of authorized pediatric products to guide all parties involved in the development and manufacturing of medicinal products in improving the availability of suitable pediatric formulations.

## 4. Modeling and Simulation in Pediatrics

The regulatory guidance documents cover various practical and ethical considerations for pediatric studies, but nevertheless, performing clinical trials in children remains a challenge and many special requirements and considerations have to be addressed up-front. Obviously, specialized tools that can help in designing and analyzing pediatric studies need to be considered and explored. 

The use of M&S methods is one possibility to support pediatric study design and to retrieve as much information as possible from data derived from trials in children.

Besides the general applicability of M&S as a beneficial methodology in drug development [71], there is a clear regulatory basis for using M&S in pediatric drug development. Several regulatory guidance documents make explicit reference to M&S techniques (see Table 8).

**Table 8:** Regulatory basis for Modeling and Simulation in pediatrics.

**International Guideline**
• ICH E11 [17] → *PK/PD modeling, population PK, and sparse sampling*
**FDA Guidelines**
• General Considerations for Pediatric Pharmacokinetic Studies [38] → *Population PK*
• Exposure-Response Relationships [60] → *Population PK and PK/PD modeling*
**EMA Guidelines**
• Concept paper on the impact of liver immaturity [51] → *Population PK and PK/PD modeling*
• Discussion paper on the impact of renal immaturity [57] → *Population PK*
• Guideline on the role of pharmacokinetics in pediatrics [44] → *Population PK and PK/PD modeling*
• Guideline on clinical trials in small populations [61] → *PK/PD modeling, Bayesian methods*
• Guideline on the investigation of medicinal products in the term and preterm neonate [45] → *PK/PD modeling, PBPK modeling, and Population PK*
• Guideline on the format and content of applications for a pediatric investigation plan [25] → *PD modeling*
• Ethical considerations for clinical trials with the pediatric population [55] → *Adaptive design*

The EMA "Workshop on modelling in paediatric medicines" (April 2008) [72] clearly shows an acceptance and endorsement of M&S techniques in pediatrics by the authorities. More research and utilization of these techniques is strongly advocated and different approaches are under discussion [69,73]. Model-based drug development in pediatrics can answer specific questions and is a success if it supports a correct decision, e.g., a good dose to use for different age groups [74].

**Figure 1:**
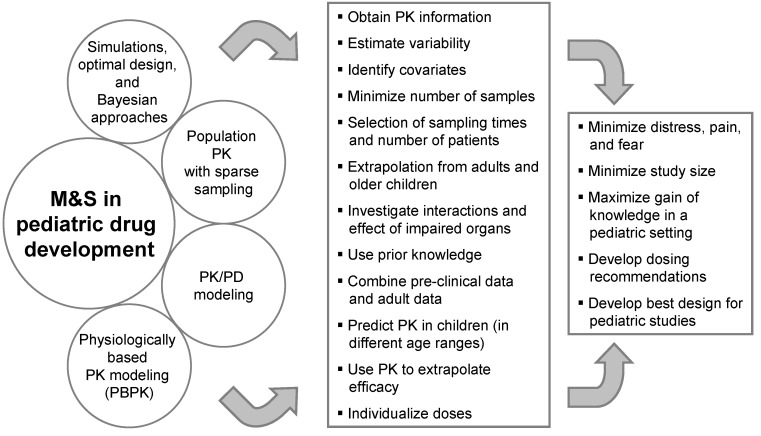
Modeling and Simulation in pediatric drug development.

Clinical trials in children are usually conduced with fewer subjects than in adults and the investigated population is a vulnerable group. Therefore, particular attention should be paid to shield them from undue risk and to minimize distress, pain, and fear. Every attempt should be made to minimize the number of participants and procedures. Therefore, the amount of information available for each subject is smaller and there is a need to be more efficient with the information/subjects at hand. Model-based drug development is of help in aiming at efficiency. M&S can help in different ways to reach this goal (see Figure 1).

The use of M&S in pediatric drug development combines two different perspectives. On the one hand, M&S tools can be applied before any pediatric data from clinical studies becomes available. Scaling approaches, PBPK modeling, Bayesian approaches making use of prior knowledge, and simulations can be used to support the pediatric strategy, e.g., for the PIP, by developing dosing recommendations and designs for pediatric studies. On the other hand, M&S techniques can be used to analyze pediatric data during and after a clinical trial. This includes sparse sampling and population PK analyses to reduce the number of blood samples and to minimize distress as well as to maximize knowledge generation and optimize further pediatric drug development with PK/PD modeling.

Since the EMA requests the PIP early in drug development, important information such as on the exposure-response relationship in adults is not yet available but early phase data can already give substantial input for M&S to support the PIP. Generally, it is almost always possible to first provide a general M&S strategy that can be later updated and/or fine-tuned with further details and results in anticipated PIP amendments.

The regulatory assessment of M&S results might differ between the U.S. and the E.U. and there is no guideline defining the requirements and acceptability. The feedback pharmaceutical companies receive after submission of PIPs or PPSRs clearly indicate that M&S techniques are endorsed or even mandatory. In addition, the establishment of the FDA Division of Pharmacometrics increased the acceptance and promotion of M&S in drug development including pediatrics. Jadhav *et al.* published a case study with an anti-hypertensive drug to show an example on how industry and FDA scientists can collaborate in designing pediatric trials using clinical trial simulations [75]. Manolis *et al.* proposed a pragmatic approach for the practical implementation of M&S in pediatrics within the current E.U. regulatory framework [73].

Population PK and PK/PD modeling, optimal design, and PBPK modeling are prominent examples of M&S techniques with an added value in pediatric drug development.

### 4.1. Population PK and PK/PD modeling in pediatrics

Population PK and PK/PD modeling are explicitly recommended in several regulatory guidelines and should be the primary analysis method in pediatric drug development (see Table 8). In particular, the FDA guideline "Exposure-Response Relationships - Study Design, Data Analysis, and Regulatory Applications" [60], outlines general considerations for PK/PD modeling and FDA perspectives for modeling strategies. Models capture the time course of drug concentration and response at the population level using mathematical stochastic equations that can be solved using a non-linear mixed effects modeling approach [76]. In addition to obtaining PK information, the variability of the PK parameters can be estimated. Population PK modeling is applicable to sparse and unbalanced data sets (neonates, children, *etc*.) and forms the scientific basis for study/trial simulations, dose adjustments or labeling extensions in other populations. 

The influence of patient-specific factors (e.g., body weight, age, sex, renal function) can be identified and tested statistically to decide whether the factor enters the population model as predictive covariate. Once a qualified model is available, it can be used for simulations, e.g., to support the selection of dosing regimen and study design. In addition, one can extrapolate from adults and older children and—if applicable—use PK information to extrapolate efficacy [77]. Depending on the age groups, empirical maturation functions and/or allometrically scaled PK parameters should be considered.

Population PK/PD models can be developed based on data from previous clinical studies or based on pooled data from different studies. This modeling technique can be combined with other information such as exposure-response relationship and disease characteristics from adults to bridge the gap between adults and children [78]. Dosing regimens based on population PK/PD models should be included in the drug label, but the model should be validated and the predictive value of the model should be sufficiently presented [79].

### 4.2. Optimal design

The evaluation and optimization of the study design becomes more and more important due to the limited number of studies, the low number of individuals and samples leading to the need of optimized and thus most informative sampling times. Prior information (e.g., PK in adults including covariates and the distribution of covariates of pediatric patients) can be employed to evaluate a sparse sampling strategy. The D-optimality sampling method allows finding a set of sampling times that yield the smallest error in the estimate of the parameters by maximizing the Fisher information matrix with respect to the sampling times. Afterwards, a large number of trials following the suggested sampling times can be simulated and the parameters can be estimated by population PK methods. These estimates can be compared to the "true" parameters used for the simulation. The comparison provides information about bias and precision to be expected in the parameter estimates if an actual study was conducted with a sampling strategy identified using the D-optimality sampling method [80,81]. An inherent risk is the assumption that relationships can be extrapolated to the patients under consideration.

### 4.3. Physiologically based PK modeling

PBPK models are based on physiological considerations and more comprehensive than empirical or semi-mechanistic models [82]. A PBPK model consists of compartments representing actual tissue and organ spaces with physical volumes of those organs and tissues. The appearance (from arterial blood) and elimination (into venous blood) of the drug in the different organs is described by mass balance equations. The model parameters include physiological and drug-specific parameters and *in vitro* predictions for distribution and elimination are utilized.

After validating a PBPK model with adult PK data, differences in growth and maturation can be accounted for to predict drug exposure in children. Since dose scaling approaches should only be used in children above two years of age, PBPK modeling can provide substantial support for pediatric dose finding in neonates and infants. Growth and maturation can affect all aspects of drug PK as well as PD and adverse effects. Physiological models for children have been established that incorporate the available knowledge and facilitate predictions of the effect of age on certain aspects of PK and PD. The performance of PBPK in describing and predicting PK in children has been proven [83,84] and several user-friendly PBPK software systems are commercially available [85,86,87]. In neonates and infants, PBPK modeling approaches are the method of choice due to the lack of reliable methods to predict drug exposure and thus dose finding in this age group. In general, PBPK models can predict drug exposure in different age groups and guide optimization of the dosing schedule and sampling times. In addition, it can support the risk assessment by investigating possible interactions and the effect of impaired organs. 

More and more pharmaceutical companies are submitting PBPK modeling reports to the EMA and the FDA and it is well possible that regulatory agencies ask for PBPK modeling approaches to support pediatric drug development in neonates and infants.

In summary, M&S can optimize the generation of knowledge in a pediatric setting, support the development of best pediatric study designs and dosing recommendations, and minimize distress, pain, and fear in pediatric patients participating in a pediatric clinical study.

## 5. Conclusions

The health authorities in the U.S. and the E.U. show a strong commitment to promote better medicines for children. The pediatric legislations have built a complex framework for pediatric drug development and the pharmaceutical industry has to deal with different requirements and special obligations to receive the incentives. The preparation of the PIP is a major task for each clinical development team and pediatric aspects have to be integrated early in development. The regulatory authorities reviewed a substantial number of pediatric evaluations in recent years and pharmaceutical companies become familiar with the pediatric regulations.

Numerous regulatory documents are available to guide pharmaceutical companies through the specific procedures and to answer specific scientific questions regarding study design and conduct. Since pediatric drug development is a very complex area, many questions remain open, and close collaboration and communication between industry and health authorities is essential.

Surprisingly, the number of companies using the free pediatric scientific advice is low compared to the number of submitted PIPs. Although the pediatric scientific advice is not binding, an open discussion about the pediatric strategy up-front can improve information exchange and reduce the time for the entire PIP procedure. Even though the guidelines cover various important aspects, the pediatric strategy is highly dependent on the properties of the drug, on the disease, and on the pediatric population and has to be defined carefully for each drug and indication. Similarly, pediatric studies vary widely and many procedural and scientific considerations (e.g., age categories, dose finding, PK sampling, pediatric formulation) are indispensable and an extraordinary challenge for each study team.

Recommended by many regulatory guidelines, M&S techniques are essential and well-established in pediatric drug development to support the pediatric strategy including dose finding, study design, and minimizing distress, pain, and fear. Recently, Gobburu [88] presented that one element of the 2020 vision of the FDA pharmacometrics group is to design all pediatric clinical trials using simulations. Jadhav and Kern [89] pointed out that the FDA is committed to channeling its pharmacometrics and clinical pharmacology resources to improve trial design and analysis of pediatric trials. They highlighted with several examples, that M&S is critical for adequately designing pediatric trials and that it has become an integral part of pediatric drug development. Accordingly, the pharmaceutical industry has to continue to evolve M&S techniques to support pediatric drug development in compliance with regulatory requirements, to jointly develop both standards and new techniques, and to improve the overall performance of the pharmaceutical industry to develop better medicines for children.
